# Does maternal oral health predict child oral health-related quality of life in adulthood?

**DOI:** 10.1186/1477-7525-9-50

**Published:** 2011-07-07

**Authors:** Dara M Shearer, W Murray Thomson, Jonathan M Broadbent, Richie Poulton

**Affiliations:** 1 Department of Oral Sciences, School of Dentistry, PO Box 647, Dunedin 9054, New Zealand; 2 Department of Preventive and Social Medicine, Dunedin School of Medicine, University of Otago, Dunedin, New Zealand

**Keywords:** oral health, oral health-related quality of life: OHIP-14, intergenerational, risk, family history

## Abstract

**Background:**

A parental/family history of poor oral health may influence the oral-health-related quality of life (OHRQOL) of adults.

**Objectives:**

To determine whether the oral health of mothers of young children can predict the OHRQOL of those same children when they reach adulthood.

**Methods:**

Oral examination and interview data from the Dunedin Study's age-32 assessment, as well as maternal self-rated oral health data from the age-5 assessment were used. The main outcome measure was study members' short-form Oral Health Impact Profile (OHIP-14) at age 32. Analyses involved 827 individuals (81.5% of the surviving cohort) dentally examined at both ages, who also completed the OHIP-14 questionnaire at age 32, and whose mothers were interviewed at the age-5 assessment.

**Results:**

There was a consistent gradient of relative risk across the categories of maternal self-rated oral health status at the age-5 assessment for having one or more impacts in the overall OHIP-14 scale, whereby risk was greatest among the study members whose mothers rated their oral health as "poor/edentulous", and lowest among those with an "excellent/fairly good" rating. In addition, there was a gradient in the age-32 mean OHIP-14 score, and in the mean number of OHIP-14 impacts at age 32 across the categories of maternal self-rated oral health status. The higher risk of having one or more impacts in the psychological discomfort subscale, when mother rated her oral health as "poor/edentulous", was statistically significant.

**Conclusions:**

These data suggest that maternal self-rated oral health when a child is young has a bearing on that child's OHRQOL almost three decades later. The adult offspring of mothers with poor self-rated oral health had poorer OHRQOL outcomes, particularly in the psychological discomfort subscale.

## Introduction

Oral-health-related quality of life (OHRQOL) measures examine the extent to which oral disease affects an individual's well-being. They aim to determine the subjective functional and psycho-social impacts of oral disease, and complement traditional objective clinical indicators to facilitate a more holistic approach to oral health [1]. Research has indicated a range of influences on oral-health-related quality of life (OHRQOL). These include direct oral health factors such as untreated caries and missing teeth [2-5], periodontal disease [2,6,7], malocclusion [5], and xerostomia [8,9]. In addition, age [3], sex [10,11], socio-economic status [2,12,13], socio-cultural factors [3,14,15], psychosocial factors [16,17], dental care services attendance pattern [12,13,18], and dental anxiety [19,20] can all impact on OHRQOL.

A potential impact on OHRQOL that remains unexamined is the effect of parental/family history of poor oral health. It is likely that intergenerational processes link maternal oral health (and maternal oral health beliefs, attitudes and behaviours) to oral health and disease risk in offspring [21-28]. If maternal oral health is associated with offspring oral health, and oral health is associated with OHRQOL, then it is reasonable to believe that maternal oral health is also associated with offspring OHRQOL through this pathway. However, is it possible that maternal oral health also exerts an influence on her offspring's OHRQOL via other mechanisms?

Population oral health and population OHRQOL, while inextricably linked, are not the same. While it can be debated whether the primary objective of public health measures should be the improvement of oral health or OHRQOL, it is likely that the burden of impaired OHRQOL is substantial. For this reason, putative predisposing factors for poor OHRQOL require careful and comprehensive examination. This will allow the identification of those who are at greatest risk of suffering poor OHRQOL due to poor oral health, but also independently of clinical oral health factors. Thus, the current study sought to determine whether the oral health of mothers of young children can predict the OHRQOL of those same children when they reach adulthood. It tested the hypothesis that mothers' self-rated oral health when children are young can predict her offspring's OHRQOL many years later.

## Methods

This study uses oral examination and interview data collected from study members during the age-32 assessment of the Dunedin Multidisciplinary Health and Development Study (DMHDS), and interview data obtained from their mothers at the age-5 assessment (in 1977/78). The DMHDS is a prospective cohort study of 1,037 children born at Queen Mary Hospital, Dunedin, New Zealand between 1 April 1972 and 31 March 1973. These 1037 children represent 91% of the 1139 eligible children born between these dates, and 972 (96% of the surviving 1015) were assessed at age 32. Ethics approval was granted by the Otago Research Ethics Committee; participants gave informed consent.

Some 922 children (88.9%) were orally examined at age 5. The accompanying parent (919 mothers, 3 fathers) was asked about dentate status and (if dentate) to rate their own oral health (responses: "excellent", "fairly good", "average", "fairly poor", or "very poor"). The three fathers were excluded from further analysis, as were six mothers who rated their oral health as "don't know". These five responses, combined with the edentulous responses, were grouped into three categories: "excellent/fairly good", "average", and "poor/edentulous", corresponding to the "excellent" or "fairly good" response; "average" response; and" fairly poor", "very poor" or "edentulous" responses respectively.

Calibrated examiners carried out dental examinations for caries and missing teeth on 932 of the 972 study members who attended the age-32 assessment (conducted November 2003-June 2005). The intraclass correlation coefficient for intraexaminer reliability was 0.99 for both examiners, and also 0.99 for interexaminer reliability; WHO examination criteria were used [29]. Tooth surfaces were examined for caries and restorations and accumulated tooth loss was also recorded, with examiners recording the reason for the absence of each missing tooth. Longitudinal caries experience data were used to identify trajectories of dental caries experience (DMFS) from age 5 to 32 (high, moderate and low), using a group-based trajectory analysis model [30]. For the purpose of performing the group-based trajectory analysis, a modified DMFS was used whereby for the 'M' component, a minimum of 3 surfaces was assigned as having been carious for each extracted tooth. This value was increased (to 4 or 5) in cases where more than 3 surfaces were known to have been carious/filled at the previous examination. This modified DMFS is preferable to the generally accepted DMFS which assumes that *every *surface of a missing tooth was decayed prior to extraction [31].

Socioeconomic status (SES) at age 32 was determined using standard NZ indices which apply a six-interval classification according to occupation; for example, a doctor scores 1 and a labourer scores 6 [32,33]. Those scoring 1 or 2 were allocated to the high-SES group; those with a score of 3 or 4 were medium-SES; and those scoring 5 or 6 were low-SES.

The short-form Oral Health Impact Profile (OHIP-14) was used to assess oral health-related quality of life [34]. This has 14 items corresponding to the seven domains outlined by Locker in his conceptual framework for assessing oral-health-related quality of life; these are functional limitation, physical pain, psychological discomfort, physical disability, psychological disability, social disability, and handicap [35]. In the OHIP-14, each subscale is represented by two items describing a problem. For each item, individuals were asked how often in the previous four weeks they had experienced the problem, responding on a five-point Likert scale. Responses were coded as "Never" (0), "Hardly ever" (1), "Occasionally" (2), "Fairly often" (3), or "Very often" (4). The total OHIP-14 score was calculated by summing the responses over all 14 items (possible scores ranging from 0-56) with a higher score indicating poorer OHRQOL. A study member was categorised as having an impact for an item if they had experienced it "Fairly often" or "Very often" in the previous four weeks. A simple count of the number of different impacts was computed (possible range 0-14). The prevalence of impacts (for subscales and overall) was defined as having experienced an impact "Fairly often" or "Very often" in the previous four weeks. The oral health characteristics of the cohort are described elsewhere, as is the distribution of responses to the individual OHIP-14 items [2]

### Statistical analysis

Descriptive and bivariate analyses used SPSS version 16.0 (SPSS Inc. Chicago, Illinois). Multivariate analyses used Stata version 11.0 (StataCorp, College Station, Texas 77845, USA). Chi-square tests examined the statistical significance of associations observed between categorical variables. Kruskal-Wallis tests were used for continuous dependent variables. *Post hoc *comparisons (using the LSD test) were conducted to determine which groups differed. Statistical tests were two-tailed (alpha = 0.05). Multivariate analysis used the generalized linear model (GLM) command with modified Poisson regression analysis (using a robust error variance procedure) to estimate the relative risk of having one or more impacts on the OHIP-14 subscales and overall scale at age 32, and to model the age-32 mean OHIP-14 score and the mean count of total number of impacts at age 32.

## Results

Of the original 1037 children, 919 (88.6%) had an oral examination at age 5, with their mother reporting on her own oral health at this assessment, and 972 (95.8% of surviving cohort) were assessed at age 32. The analysis was limited to the 827 (81.5% of surviving cohort) study members who were dentally examined at both ages, who completed the OHIP-14 at the age-32 assessment, and whose mothers had been interviewed (and self-rated their oral health or were edentulous) at the age-5 assessment. An attrition analysis found statistically significant differences between those who were examined at both ages, and those who were examined at age 5 only (Table 1). The latter were more likely to be low SES, and to have a mother who self-rated her oral health as poor, or was edentulous, at the age-5 assessment, than the former. The likely effect of these differences is attenuation of the associations.

**Table 1:** Comparison of study members who attended with their mother at the age-5 assessment who were examined, and completed OHIP-14, at age 32, versus those who were not examined, or did not complete OHIP-14, at age 32

	Examined and completed OHIP-14 at age 32 (N = 827)	Not examined or did not complete OHIP-14 at age 32 (N = 86)
**Study members at age 5 assessment**		
Male (%)	419 (50.7)	49 (57.0)
SES from birth to age 15^1^		
High (%)	130 (15.8)^a^	6 (7.1)
Medium (%)	529 (64.2)	53 (62.4)
Low (%)	165 (20.0)	26 (30.6)
Caries-free at age 5 (%)	335 (40.5)	39 (45.3)
Mean dmfs at age 5 (SD)	3.7 (5.6)	3.3 (4.9)
**Mothers at age 5 assessment**		
Self-rated oral health as fairly poor/very poor or was edentulous (%)	238 (28.8)^b^	38 (44.2)

^1^Not all participants could be classified for the SES from birth to age 15 variable; ^1^N = 909

^a^p < 0.05; chi-square test. ^b^p < 0.01; chi-square test.

Associations were found between mothers being edentulous or having poor/fairly poor self-rated oral health status at the age-5 assessment and the prevalence of an OHRQOL impact (as measured by a "fairly often" or "very often" response to OHIP-14 scale items) among study members at age 32 (Table 2). Unadjusted bivariate associations between study members' OHIP-14 impacts at age 32 and maternal self-rated oral health 27 years earlier revealed a gradient of higher prevalence of impacts for the overall scale by poorer maternal self-rated oral health/edentulous status at the age-5 assessment. Mothers who rated their oral health as poor (or who were edentulous) at the age-5 assessment had offspring with higher mean total OHIP-14 score at age 32, and higher mean count of total number of impacts at age 32, than did mothers who did not rate their oral health as poor, and were dentate.

**Table 2:** Study members' oral health-related QOL at age 32 by mothers' self-rated oral health status at the age-5 assessment (an impact is measured by a "fairly often" or "very often" response to OHIP-14 scale items)

	Mother self-rated oral health at age 5 assessment			
	
	Excellent or fairly good (N = 271)	Average (N = 318)	Fairly poor, very poor or edentulous (N = 238)	Total (827)
**Age 32 OHRQOL (OHIP-14 scale)**				
**Prevalence 1+ impacts (%)**				
1+ impacts overall	54 (19.9)^a^	68 (21.4)	75 (31.5)	197 (23.8)
**Severity of impacts (SD)**				
Mean total OHIP score	6.6 (6.9)^bc^	8.4 (8.0)^b^	9.3 (8.9)^c^	8.1 (8.0)
Mean count of total number impacts	0.4 (1.0)^d^	0.5 (1.2)^e^	0.8 (1.8)^de^	0.5 (1.4)

^a^p < 0.005; chi-square test

^b, c, d, e ^p < 0.005 Kruskal-Wallis test; Estimates with different symbols are significantly from each other (by *post hoc *criteria)

Multivariate modelling was used to determine the relative risk (RR) of having one or more impacts on the OHIP-14 scale, and on each of the subscales, at age 32, for each of the categories of maternal self-rated oral health at the age-5 assessment (using the "excellent/fairly good" category as a referent), while controlling for low SES, high caries trajectory, and having one or more missing teeth. There was a consistent gradient of RR across the categories of maternal self-rated oral health status at the age-5 assessment for having one or more impacts in the overall OHIP-14 scale, whereby risk was greatest among the study members whose mothers rated their oral health as "poor/edentulous", and lowest among those with an "excellent/fairly good" rating (Table 3). Regarding the subscales, the only finding to reach statistical significance was the "excellent/fairly good" category in the psychological discomfort subscale (Table 4). The risk of a study member whose mother had rated her oral health as "poor/edentulous" of suffering an impact in the psychological discomfort subscale at age 32 was over double that of a study member whose mother had rated her oral health as "excellent/fairly good".

**Table 3:** Adjusted* estimates for study members' relative risk of having 1+ impacts on overall OHIP-14 score, mean OHIP-14 score, and mean number of different OHIP-14 impacts at age 32, by maternal self-rated oral health 27 years earlier (modified Poisson regression model)

	Study members' relative risk of 1+ impacts on overall OHIP-14 score by age 32 (95% CI)		Study members' mean OHIP-14 score by age 32 (95% CI)		Study members' mean number of different OHIP-14 impacts by age 32 (95% CI)	
**Mother's self-rated oral health 27 years earlier**						
Excellent/fairly good	1.00		6.77	(6.46, 7.09)	0.35	(0.28, 0.42)
Average	1.02	(0.75, 1.40)	8.24	(7.93, 8.56)	0.44	(0.37, 0.51)
Fairly poor, very poor or edentulous	1.27	(0.93, 1.74)	8.14	(7.78, 8.51)	0.59	(0.50, 0.69)

*Controlling for low SES, high caries trajectory, 1+ missing teeth.

**Table 4:** Modified Poisson regression model for study members' prevalence of one or more impacts on OHIP-14 scale subsets, and prevalence of one or more impacts on OHIP-14 overall scale at age 32

	Low SES at age 32	High caries trajectory	1+ missing teeth	Mother self- rated oral health as average	Mother self- rated oral health as poor/edentulous
**1+ impacts on OHIP-14 subscales and overall** ** scale at age 32**					
**RR (95% confidence intervals)**					
1+ impacts - overall (any subscale)	**1.45 (1.14, 1.85)**	1.28 (0.96, 1.72)	**1.89 (1.45, 2.44)**	1.02 (0.75, 1.40)	1.27 (0.93, 1.74)
1+ impacts - *functional limitation *	1.61 (0.73, 3.56)	1.72 (0.60, 4.94)	1.91 (0.74, 4.94)	1.18 (0.32, 4.32)	2.72 (0.84, 8.75)
1+ impacts - *physical pain *	1.02 (0.61, 1.72)	**2.06 (1.18, 3.62)**	**2.47 (1.43, 4.27)**	1.48 (0.73, 3.04)	1.71 (0.87, 3.36)
1+ impacts - *psychological discomfort *	1.31 (0.87, 1.99)	1.50 (0.91, 2.48)	**2.62 (1.65, 4.16)**	1.49 (0.83, 2.66)	**2.08 **(**1.16, 3.72**)
1+ impacts - *physical disability *	1.50 (0.99, 2.28)	1.17 (0.72, 1.91)	**1.77 (1.15, 2.70)**	0.90 (0.56, 1.45)	0.90 (0.54, 1.49)
1+ impacts - *psychological disability *	1.09 (0.63, 1.89)	1.80 (0.98, 3.31)	**2.40 (1.35, 4.27)**	1.36 (0.66, 2.82)	1.49 (0.71, 3.11)
1+ impacts - *social disability*	1.90 (0.84, 4.28)	0.63 (0.23, 1.76)	**2.89 (1.30, 6.42)**	0.97 (0.36, 2.59)	1.85 (0.75, 4.47)
1+ impacts - *handicap *	1.83 (0.76, 4.37)	1.14 (0.36, 3.64)	2.15 (0.85, 5.47)	0.54 (0.19, 1.54)	0.81 (0.29, 1.54)

Reference categories: low SES at age 32 (high/medium SES coded 0), high caries trajectory (low caries trajectory coded 0), 1+ missing teeth at age 32 (no missing teeth coded 0), Mother self-rated oral health as average (Mother self-rated oral health as excellent coded 0), Mother self-rated oral health as poor/edentulous (Mother self-rated oral health as excellent coded 0)

RR, relative risk; SES, socioeconomic status.

Using a modified Poisson approach, the study members' mean OHIP-14 score at age 32, and mean number of OHIP-14 impacts at age 32, were modelled for each of the three categories of maternal self-rated oral health at the age-5 assessment, while controlling for low SES, high caries trajectory, and having one or more missing teeth. There was a gradient in the age-32 mean OHIP-14 score across the categories of maternal self-rated oral health status at the age-5 assessment, whereby the study members whose mothers self-rated their oral health as "average" or "poor/edentulous" at the age-5 assessment had higher mean OHIP-14 scores (and thus poorer OHRQOL) than those whose mothers self-rated their oral health as "excellent/fairly good" (Table 3). There was also a clear gradient in the mean number of OHIP-14 impacts at age 32 across the three categories, whereby it was greatest among the study members whose mothers rated their oral health as "poor/edentulous", and lowest among those whose mothers rated their oral health as "excellent/fairly good". The difference between the "poor/edentulous" group and the "excellent/fairly good" group was statistically significant. The differences between the "poor/edentulous" group and the "average" group, and between the "excellent/fairly good" group and the "average" group, did not reach statistical significance, however.

## Discussion

This is the first time that intergenerational associations between parental oral health and OHRQOL in offspring have been examined. These prospective cohort study data suggest that maternal self-rated oral health when a child is young has a bearing on that child's OHRQOL almost three decades later. The adult offspring of mothers with poor self-rated oral health had poorer OHRQOL outcomes, particularly in the psychological discomfort subscale.

This study had some limitations. The group included in the analysis were those who were examined at both ages, and who also completed the OHIP-14 questionnaire at age 32. This group differed in some ways from those examined only at age 5 (and were not included). More of the latter were low SES, and had a mother who was edentulous or had poor oral health in 1977/78. This may have led to an under-estimation of the strength of the observed associations. Although data were available for a wide range of potential confounders, it was not practical to control for all of these in the models. Those which were included were chosen on the basis of biological plausibility, and guided by previous research. We relied on maternal self-report data at the age-5 assessment, and on study members' self-report data on SES at the age-32 assessment. The reliability and validity of self-report data has been addressed elsewhere [36]. In the case of the Dunedin Study, self-report data are gathered by interview/examiner-based assessments rather than self-completed questionnaires, and so are more likely to yield valid data. In addition, study members (and their parents) are aware of the importance of accurate responses, and there is a long history of mutual trust and respect between study members and researchers. To date, it has not been possible to examine the nature and extent of such cross-generational associations for OHRQOL because the required data have not been available. The strengths of the Dunedin Study are its longevity, sample size, retention rate, detailed oral health data, and information on a range of potential risk, ameliorating, exacerbating and confounding factors. In addition to this unique dataset, data were also collected from the mothers when their children were young. This provided a rare opportunity to investigate intergenerational associations in oral health, and to broaden understanding of the possible causal associations between the oral health, attitudes and beliefs of mothers of young children and OHRQOL of these children many years later. The use of a birth cohort--and the high retention rate--means that the sample is broadly representative of its source population (New Zealand's South Island). Whether the findings can be generalized to the New Zealand population, and to other populations (particularly the United States), has been addressed in earlier work [37], where it was concluded that oral health findings from the DMHDS can cautiously be generalized to these populations.

While these findings are unique, they are moderately consistent with data on adults that are available from other studies, thus increasing confidence in the validity of the longitudinal and intergenerational findings [3,6,12,14,16]. The mean OHIP-14 score in the DMHDS sample was higher than that found in samples from Australian populations [3,12,14,16]; it was also markedly higher than in British and German populations [3,14,38,39], but lower than in a Chinese sample [6]. In addition, the prevalence of impacts was greater in the DMHDS sample than in the British and Australian samples [12,14]. It should be noted that, while these studies sampled a range of ages, the Dunedin Study members, at age 32 years, were likely to report worse OHRQOL than any other age group reported upon to date [3]. While it is unclear why the DMHDS scores are generally higher than other population samples' scores, it is likely to be partly due to age and cultural background, as these have been found to be important variables influencing OHRQOL [3]. Moreover, the exceptionally high participation rate of the DMHDS means that there is the full range of pathology present, and this would tend to make the scores higher than those seen in samples with lower participation rates. The findings for the mothers at the age-5 assessment agree with the findings of the New Zealand Survey of Adult Oral Health carried out in New Zealand during 1976, and it is reasonable to assume that the mothers were representative of their generation [40].

The longitudinal associations found between maternal self-rated oral health at the age-5 assessment and study members' OHRQOL at age 32 were noteworthy, with clear gradients in the mean OHIP-14 score, the mean total number of impacts, and the prevalence of one or more impacts overall. Findings from a recent study provide evidence of a strong association between maternal self-rated oral health when children are young, and caries risk among those children many years later [41]. Children of mothers with unfavourable self-rated oral health had a greater risk of having a high caries trajectory--and of having a higher DMFS score--almost three decades later, than children of mothers with better self-rated oral health. It is likely that the OHRQOL gradients observed across the categories of maternal self-rated oral health are partly mediated by poorer oral health in the study members [2-5]. In this sample, the strongest predictor of the prevalence of one or more impacts in the OHIP-14 overall scale, and in most of the subscales, is having one or more missing teeth. However, the findings in the mean OHIP-14 score, and with the psychological discomfort subscale were independent of caries trajectory and the presence of missing teeth. SES is also generally considered to be a mediator of differences in OHRQOL between groups as (a) SES has been shown to influence OHRQOL (and very probably influenced maternal self-rated oral health 27 years before), and (b) SES in one generation is associated with SES in the next [2,12,13,42-44]. Nonetheless, our findings for the mean OHIP-14 score and psychological discomfort subscale were independent of low SES. The effect sizes of the associations found are small (for example, Cohen's d was 0.3 for the mean total OHIP-14 score) [45]; and the difference in adjusted mean OHIP-14 score between the "excellent/fairly good" category and the other two categories is less than one-quarter of a standard deviation [46]. However, these associations have endured over a long time, and consequently they are important.

There has been some interesting debate in recent years on the dimensional structure of the OHIP-14 scale, with the suggestion of the existence of a more parsimonious set of OHIP dimensions comprising *psychosocial impact*, *orofacial pain*, *oral functions *and *social impact *[12,47,48]. It has been proposed that the OHIP-14 functional limitation dimension may correspond to the *oral functions *dimension; the physical pain dimension to the *orofacial pain *dimension; the psychological discomfort dimension to the *social impact *dimension; and the physical disability, psychological disability, social disability, and handicap dimensions to the *psychosocial impact *dimension [38]. Factor analysis/principal components analysis to explore the dimensional structure of the OHIP-14 scale in the DMHDS cohort would be of value in examining this issue (although this is beyond the scope of this paper).

Of the prevalence of impacts within the individual subscales, only the psychological discomfort subscale (or the "*social impact" *scale according to a reduced set of subscales) gradient showed an association which was independent of confounders. Is there a reason why this one subscale should be the only one independently associated with maternal self-rated oral health from 27 years earlier? While it is unclear exactly how mothers' self-rated oral health impacts on their adult offspring's OHRQOL 27 years later, it is likely there are complex psychological, socioeconomic and socio-cultural factors processes at play, as well as the more direct diffusion of oral health status (and oral health behaviours) between the generations. The psychological discomfort subscale asks two questions of study members: how often in the previous four weeks they had (a) been self-conscious, or (b) had felt tense, because of trouble with their teeth, mouth or dentures, responding on the five-point Likert scale. While mothers' response to the self-reported oral health question asked at the age-5 assessment undoubtedly reflected their physical oral health at that time, it is possible that it also captures another domain involving psychosocial factors--one similar to that captured by the psychological discomfort subscale--in effect, "negative emotionality". Negative emotionality is one of the three superfactors of the Multidimensional Personality Questionnaire; it comprises aggression, alienation and stress reaction subscales, and a high score is related to anger, depression, neuroticism, anxiety, and poor reaction to stress [49]. In addition, research has linked negative emotionality with poorer self-reported oral health, whether measured by the OHIP-14 or by a single-item global measure [50]. It is plausible that negative emotionality influenced some mothers' response to the self-rated oral health question at the age-5 assessment, and that negative emotionality was passed to her child. This in turn had an effect on the OHIP-14 psychological discomfort subscale in that child 27 years later. In any case, what is clear is that longitudinal associations between maternal self-rated oral health and adult offspring OHRQOL are likely to be the result of an intricate combination of genetic predisposition coupled with exposure to environmental risk factors, including direct oral health factors, oral health behaviours, socioeconomic, socio-cultural and psychosocial effects.

## Conclusions

Severe oral disease is now concentrated among a relatively small group of individuals. The targeting of those most at risk of both oral disease, and the subjective impact of oral disease, will ensure the most efficient use of scarce resources. This evidence suggesting that OHRQOL can be influenced by circumstances early in the life course means the *when*, and for *whom*, of preventive interventions must be carefully considered. As far as effective intervention is concerned, later may be simply too late. Intervention early in the life-course is essential. As for *whom*, those children whose mothers (and these days, fathers) rate their own oral health unfavourably must be considered to be at greater risk than most.

## Competing interests

The authors declare that they have no competing interests.

## Authors' contributions

DMS participated in the data analysis and interpretation, and in drafting the manuscript. WMT participated in the conception and design of the study, and in drafting the manuscript. JMB made critical comments on the manuscript, and participated in drafting the manuscript. RP made critical comments on the manuscript, and helped to draft the manuscript. All authors read and approved the final manuscript.
